# A Global Collaboration to Develop and Pilot Test a Mobile Application to Improve Cancer Pain Management in Nepal

**DOI:** 10.3389/fpain.2022.910995

**Published:** 2022-07-28

**Authors:** Virginia T. LeBaron, Bethany J. Horton, Abish Adhikari, Sandhya Chapagain, Manita Dhakal, Rajesh Gongal, Regina Kattel, Ganesh Koirala, Anna Kutcher, Ben Hass, Martha Maurer, Daniel Munday, Bijay Neupane, Krishna Sharma, Ramila Shilpakar, Amuna Shrestha, Sudip Shrestha, Usha Thapa, Rebecca Dillingham, Bishnu D. Paudel

**Affiliations:** ^1^University of Virginia School of Nursing, Charlottesville, VA, United States; ^2^University of Virginia School of Medicine, Charlottesville, VA, United States; ^3^Kathmandu Cancer Center, Tathali, Nepal; ^4^National Academy of Medical Sciences, Bir Hospital, Kathmandu, Nepal; ^5^B.P. Koirala Memorial Cancer Hospital, Bharatpur, Nepal; ^6^Hospice Nepal, Kathmandu, Nepal; ^7^Nepal Cancer Hospital & Research Center, Lalitpur, Nepal; ^8^Hass Software Consulting, Brooklyn, NY, United States; ^9^Sonderegger Research Center, University of Wisconsin School of Pharmacy, Madison, WI, United States; ^10^Usher Institute, University of Edinburgh, Edinburgh, United Kingdom; ^11^University of Virginia Center for Global Health Equity, Charlottesville, VA, United States

**Keywords:** palliative care, cancer, mobile health, mobile applications, pain, pain management guidelines, Nepal, capacity building

## Abstract

**Introduction:**

Quality palliative care, which prioritizes comfort and symptom control, can reduce global suffering from non-communicable diseases, such as cancer. To address this need, the Nepalese Association of Palliative Care (NAPCare) created pain management guidelines (PMG) to support healthcare providers in assessing and treating serious pain. The NAPCare PMG are grounded in World Health Organization best practices but adapted for the cultural and resource context of Nepal. Wider adoption of the NAPCare PMG has been limited due to distribution of the guidelines as paper booklets.

**Methods:**

Building on a long-standing partnership between clinicians and researchers in the US and Nepal, the NAPCare PMG mobile application (“app”) was collaboratively designed. Healthcare providers in Nepal were recruited to pilot test the app using patient case studies. Then, participants completed a Qualtrics survey to evaluate the app which included the System Usability Scale (SUS) and selected items from the Mobile App Rating Scale (MARS). Descriptive and summary statistics were calculated and compared across institutions and roles. Regression analyses to explore relationships (α = 0.05) between selected demographic variables and SUS and MARS scores were also conducted.

**Results:**

Ninety eight healthcare providers (*n* = 98) pilot tested the NAPCare PMG app. Overall, across institutions and roles, the app received an SUS score of 76.0 (a score > 68 is considered above average) and a MARS score of 4.10 (on a scale of 1 = poor, 5 = excellent). 89.8% (*n* = 88) “agreed” or “strongly agreed” that the app will help them better manage cancer pain. Age, years of experience, and training in palliative care were significant in predicting SUS scores (*p*-values, 0.0124, 0.0371, and 0.0189, respectively); institution was significant in predicting MARS scores (*p* = 0.0030).

**Conclusion:**

The NAPCare PMG mobile app was well-received, and participants rated it highly on both the SUS and MARS. Regression analyses suggest end-user variables important to consider in designing and evaluating mobile apps in lower resourced settings. Our app design and pilot testing process illustrate the benefits of cross global collaborations to build research capacity and generate knowledge within the local context.

## Introduction

An estimated 60 million people in the world have unmet palliative care needs, the majority of whom live in low and middle-income countries (LMICs) (1). Delivering quality palliative care, which prioritizes comfort and symptom control—especially optimal pain management—can reduce global suffering from non-communicable diseases (NCDs), such as cancer (2–5). For the purposes of this paper palliative care is broadly defined as holistic care provided to patients (and their family caregivers) with serious illness that has comfort and symptom control as the primary goal. Palliative care is particularly important in LMICs, where the cancer burden is rapidly growing, and where the majority of patients are first diagnosed with late stage cancer, which has a poor prognosis and is commonly accompanied by serious pain (6–8). Effectively managing physical pain is a foundational principle of palliative care (9), yet pain remains **one** of the most common, and most feared, symptoms of cancer (10–13). The World Health Organization (WHO) estimates that over 5.5 million people worldwide receive no, or minimal, treatment for their cancer pain (5, 14). In 2014 the World Health Assembly passed the landmark Palliative Care Resolution, which officially urged Member States to integrate palliative care in to national healthcare systems (15). Basic palliative care and pain relief has also been declared a universal human right (16–19). Despite these imperatives, a 2015 survey of 21 healthcare institutions in Nepal revealed that 83% of Nepal's population does not receive adequate palliative care (20).

Multiple professional organizations have created pain management guidelines (PMG) to address knowledge gaps and help healthcare providers (HCPs) effectively manage cancer pain (21–25). Unfortunately, despite evidence suggesting clear benefits, adherence to PMG remains low (26–29), and understanding contextual barriers to low adherence of PMG is limited, especially in LMICs (11, 27). One challenge is that cancer care guidelines are often “imported” from higher-resource, Western-oriented settings and may not translate well to LMIC settings. To address these challenges, it is critical that guidelines are designed and created that are relevant for the LMIC context. Once culturally and contextually relevant guidelines are created, leveraging Mobile Health (“mHealth”)—the use of mobile/wireless technology to improve healthcare—may enhance PMG implementation and adherence (30, 31). One viable approach to improve adherence to PMG and cancer pain care is *via* Mobile Health (“mHealth”) (32). mHealth (a subfield of electronic or “e-health”) involves leveraging mobile/wireless technology to improve healthcare (33). mHealth interventions range from 1-way text message alerts to mobile applications or “apps”—software programs downloaded to a mobile device (e.g., smart phone). A benefit of apps is that they are dynamic and portable, and can be accessed wherever, and whenever, the user desires (34). mHealth is increasingly viewed as a viable strategy in LMICs due to exploding telecommunication network capacity and decreasing cost of mobile devices (33, 35–41). For example, 73% of the 5.3 billion global mobile phone users are located in LMICs, with increasing penetration in remote rural areas (37). There is growing evidence that mHealth in LMICs can increase access to care (30, 33), support frontline healthcare workers (37, 39, 42, 43), enhance data collection (31, 44), and improve patient outcomes (45–47), but finding ways to evaluate effectiveness can be challenging (48, 49).

Nepal is a LMIC with a growing cancer burden and is well-poised to engage in collaborative palliative care and mHealth research. NCDs, including cancer, account for almost 60% of deaths in Nepal and are considered a major public health issue (50–55). In 2012, an estimated 58,000 adults needed palliative care in Nepal; approximately 20% were adults with cancer. These are likely underestimates, however, as Nepal only recently developed a population-based national cancer registry program (56–58), and does not yet have a national cancer control plan (56). Nepal does, however, have a national multisectoral NCD plan that recognizes the need for improved cancer services, including palliative care (59). The Nepal NCD plan also specifically calls to increase research capacity by strengthening infrastructure and training investigators (56, 59). A challenge in palliative care, particularly in LMICs, is generating and implementing evidence based research (60–63). Tremendous progress has been made over the past 5–10 years in Nepal to increase societal and healthcare provider (HCP) awareness regarding palliative care, to increase opioid availability for pain relief, and to understand the scope of palliative care needs (20, 56, 64, 65). However, gaps remain related to the infrastructure needed to conduct rigorous research. Research has been proposed as the fifth pillar of the WHO Public Health Strategy for Palliative Care (66), yet little palliative care research is conducted in LMICs (60, 62, 63, 66, 67).

This research represents a history of collaborative partnerships and successful initiatives undertaken in Nepal to improve palliative care and pain relief. Importantly, it also leverages foundational in-country work of The Nepalese Association of Palliative Care (NAPCare). NAPCare, a non-political, non-government organization, was founded in 2009 by community advocates and clinicians to improve access to palliative care (68). NAPCare has worked tirelessly to advance palliative care services and improve availability of essential pain medicines for patients with cancer (20, 64, 69), including the creation of Palliative Care Pain Management Guidelines (PMG) (70). First drafted in 2011, and updated in 2017, the NAPCare PMG are based on the WHO analgesic ladder (71) and adapted to the Nepal context. The NAPCare PMG have been distributed as paper booklets and designed to guide clinicians in appropriate cancer pain management. To date, implementation of the PMG has been limited.

With funding support from the National Institutes of Health, Fogarty International Center, US researchers partnered with investigators in Nepal to co-create and pilot test the “NAPCare PMG mobile health app,” which transformed the original paper-based PMG into a mobile app for use by healthcare providers (HCPs) (72). Our interdisciplinary team representing nursing, medicine, and social work consisted of clinicians and researchers from Nepal, United Kingdom, and the United States with expertise in oncology/palliative care, pain management, global health, and development and testing of mobile applications to improve health outcomes. Importantly, this research leveraged the enthusiasm, commitment, existing palliative care clinical services, and outstanding human capital within Nepal to conduct quality research that can inform and advance the evidence base regarding mHealth. The broad, overarching goal of this work is to support healthcare providers in delivering quality cancer pain care in LMICs, build research capacity within Nepal, and, ultimately, decrease patient suffering related to under or untreated cancer pain. A key objective of this paper is to present our collaborative process of mobile app development in sufficient detail so that it may be helpful to other researchers engaging in similar global work.

## Materials and Methods

### Overall Design

This was a feasibility and acceptability study grounded in a Community Based Participatory Research (CBPR) philosophy to develop and pilot test a mHealth decision support application “app” to promote PMG implementation within Nepal. CBPR is a collaborative research approach that involves engaging community stakeholders as members of the research team to implement relevant, sustainable change (73–76) and is a constructive approach in LMICs and can bridge knowledge and action, give voice to marginalized communities, and reduce health inequities (73–76). Core CBPR principles relevant to this research include engaging local stakeholders in establishing research priorities and the design of the project, recognizing and capitalizing on local expertise, sharing collective knowledge to work toward positive change, and promoting leadership growth and autonomy within the community (77). CBPR is a well-established research method and advocated as a critical approach to successful mHealth initiatives (33, 78–80).

### Design of the NAPCare PMG Mobile Application

The design and pilot testing of the NAPCare PMG mobile app was a highly iterative, collaborative process between the Nepal and UVA research team facilitated by in-country fieldwork and remote virtual meetings and focus groups that occurred between November 2019—February 2021. Our initial fieldwork prompted important questions related to the scope, intent, and distribution of the mobile app, which were critical to discuss at the onset to clarify expectations. For example, there was significant interest by Nepal team members for the app to facilitate communication across healthcare providers within an institution, track patients over time, and link app results with existing clinical information documented in the medical record. However, the institutions selected for pilot testing generally lacked an existing electronic health record and building out such an infrastructure was well beyond the scope of this feasibility study. Key considerations, questions and challenges related to the NAPCare mobile app design and development are summarized in Table 1.

**Table 1:** Key considerations related to the NAPCare mobile app design and development.

• How our prior needs assessment survey (81) could best inform app design.
• Confirming target audience and patient population for app.
• How to balance a thorough pain assessment with an easy, quick user interface.
• Deciding what demographic data to collect from healthcare providers and patients.
• Questions regarding data sharing, security, and intellectual property questions (e.g., who will “own” and maintain the app and the data collected after it is designed?)
• Staying “true” to the NAPCare PMG while recognizing that there is often more than one “right” answer or approach to managing cancer pain.
• How to best gather feedback about the app from a diverse, global team.
• Importance of ensuring clinical accuracy and safety of app recommendations.
• Tailoring features to the needs of different types of healthcare providers.

The app design process was grounded in an Information Systems Research (ISR) framework, which advocates an iterative and collaborative process, focused on user needs (82). Our first step was to share the paper version of the NAPCare PMG with the engineering team to familiarize them with the document and discuss the goals and intent of the app. Then, a first draft of wireframes and high-fidelity app screen mock-ups were created and shared with study team members over Zoom and via Google docs to gather feedback and make iterative changes. In January 2020, an interactive, in-person workshop was held in Nepal to discuss app development. The focus of these discussions was to get the “foundation of the house” right (i.e., ensure the underlying architecture of the app is accurate) versus worrying too much about “what color we are painting the walls” (i.e., aesthetic details that are relatively easy to change). We discussed and clarified important topics, such as how to balance a thorough pain assessment with an easy, quick mobile app user interface recognizing that “we could build a ‘great' app but if no one uses it, it's useless.” We also clarified the intended audience for the app (nurses and physicians) and patient population (adult patients with cancer pain). Team members also wrote down on sticky notes “need,” “nice” and “next” preferences for the app. An example of the “need, nice, next” exercise was given to start: “*I need a new motorcycle. A “need” is that it runs and can get me from point A to point B. A “nice” is that is has good gas mileage or that the color is red. A “next” is that it has Bluetooth capability so I can listen to my music while I ride*.” Team members then put their sticky notes up on the wall in the “need” “nice” and “next” columns. The lists were read and discussed to reach group consensus regarding which preferences belonged in which columns and to inform app design (Table 2).

**Table 2:** Desired features and functionality of the NAPCare mobile app as identified by the Nepal team.

**Completed with this current study (“Need”)**	**Planned for next project (“Nice,” short term)**	**Future (“Next,” long term)**
• Fast; provider can go through app quickly, especially for patients in severe pain.	• Enhanced information regarding opioids and adjuvants for each type of pain.	• Pediatric dosing and pain management guidelines.
• Able to support patients with mixed types of pain. • Off-line use; does not require Internet connectivity to use app or enter data. • Includes basic information regarding opioids and adjuvants for each type of pain. • Includes information on types of pain and pain scale assessment information. • Easy to navigate; user friendly; intuitive. • Free and accessible. • Visually appealing; not cluttered or confusing. • App works properly; when you click on something it takes you to the right place/link. • Useful for physicians and nurses, especially new learners.	• Opioid dose calculation conversion tool. • Links to tools and additional palliative care and pain management related resources. • Easily accessible to many people (i.e., scale-up app distribution to large number of generalist cancer care HCPs^*^.) • Ensure medical accuracy/safety of app recommendations through enhanced testing and regular clinical reference review. • More warning toasts/alerts and key reminders related to medication selection, side effects, contraindications, severe pain, and timing for patient reassessment. • Trigger for consult to palliative care/pain specialist. • Pain selector; more specific body pain locator. • Educational components that HCP user can share with patients and/or family members. • Add additional skip logic methodology based on data entered by the HCP to create an even more dynamic app decision support interface.	• How to track same patient visits in the same center (e.g., syncing patient info across devices). • Can share information/data among fellow HCPs. • Management of opioid overdose/monitoring and management. • Follow-up checklists/reminders (e.g., physical exam and labs). • Collects and tracks patient reported symptoms and distress levels. • Automatically updates with new medications and guidelines. • How to find strong opioids/supply indicator; helps HCPs know where to refer patients so they can get opioid prescriptions filled. • Supports complex medication decisions (e.g., in case of renal failure and altered liver function). • Is “smart” enough to learn what patient needs and guides HCP.

* *HCP, healthcare provider*.

**Figure 1:**
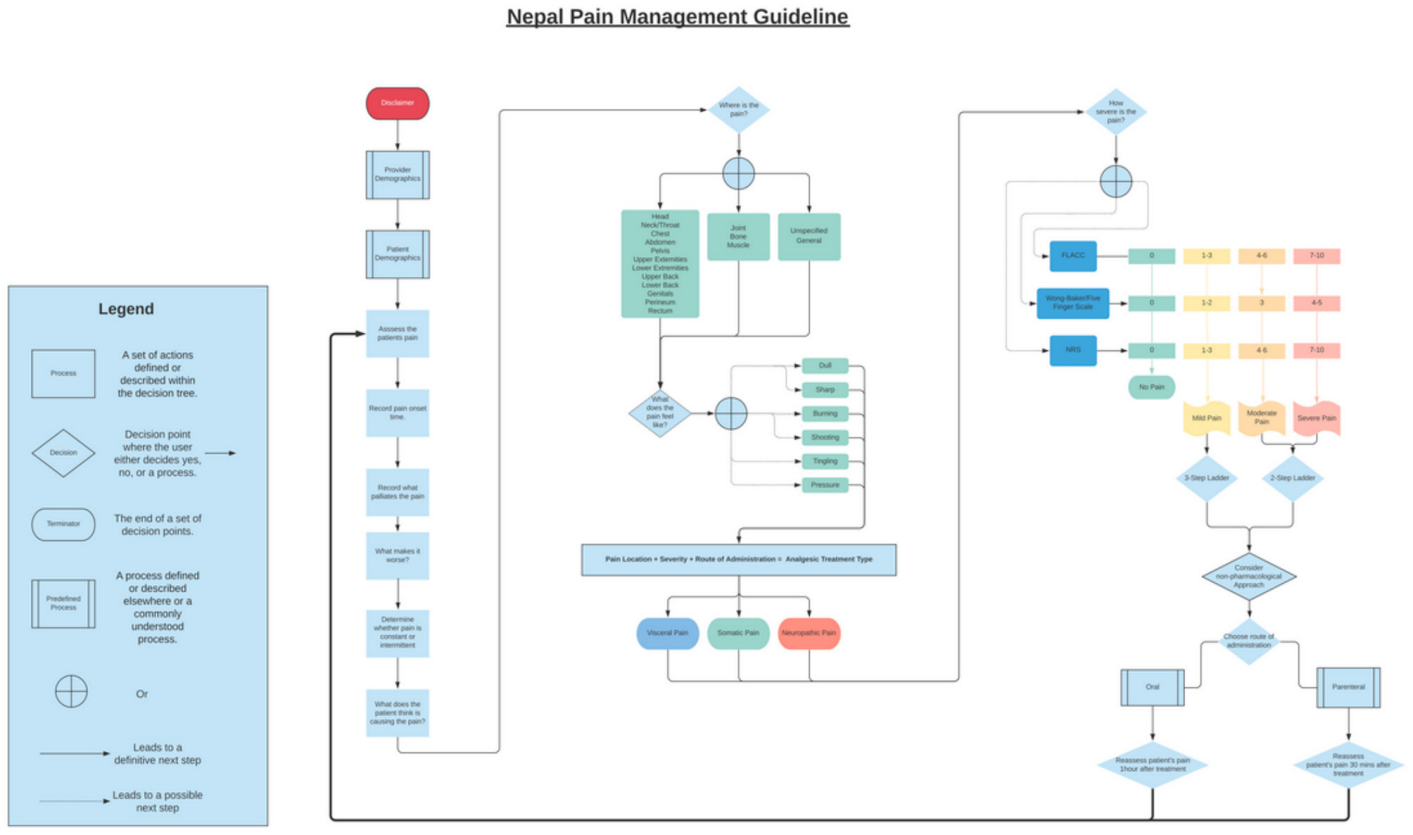
An example of a pre-defined process flowchart used to inform the NAPCare PMG app design.

**Figure 2:**
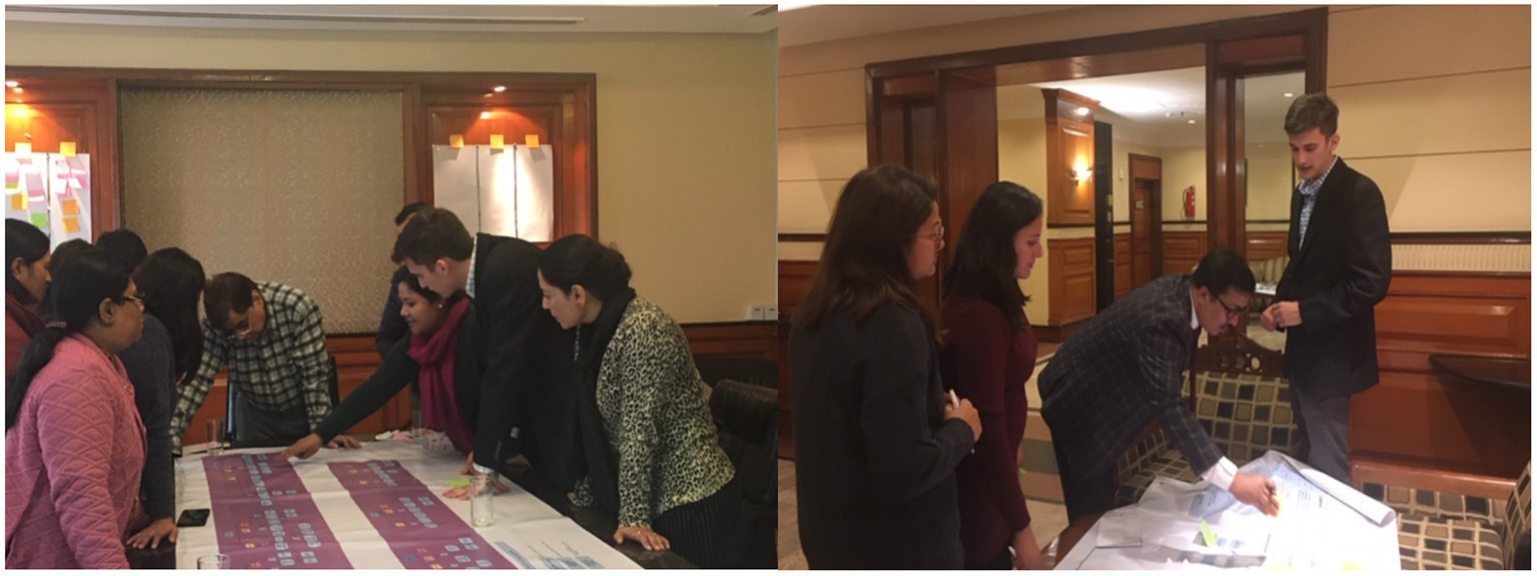
Discussing pre-defined process flowcharts to inform app design with the Nepal team during a fieldsite visit.

A next step was to convert the NAPCare PMG into pre-defined processes that could inform the design and programming of the mobile app (Figure 1). Working collaboratively, the Nepal and UVA team iterated various flowcharts of pre-defined processes related to the NAPCare PMG. During the in-person field visit to Nepal, members of the UVA team printed out large, 3′ × 6′ colored, vinyl posters of the most updated pre-defined process flowcharts. The posters were displayed, and team members circulated around the posters and used different colored sticky notes to make suggestions and comments directly on the decision flowchart posters. We validated and finalized these processes by testing various simulated patient case scenarios to ensure the pre-defined process algorithms produced the correct results. Specifically, Nepal team members brainstormed 5 common pain scenarios they see in clinical practice and worked through these cases on the decision flowchart posters. Each clinical decision point in the pain assessment and treatment process was marked with a sticky note. This was a very helpful exercise and allowed the team to identify problems with flow in the app, or decisions that needed to come earlier, later or were unnecessary (Figure 2).

**Figure 3:**
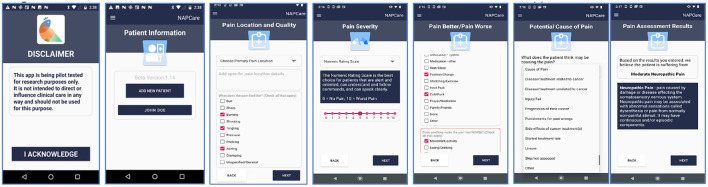
Selected screenshots of the beta version of the NAPCare PMG mobile health app 1.0.

After the in-person workshop in Nepal, additional rounds of iterative feedback were conducted remotely using GoogleDocs to finalize the app decision flowcharts and create high-fidelity mock-ups of the various app screens. The mock-ups were reviewed, discussed, and iteratively revised during regular, collaborative meetings between the Nepal and UVA teams held over Zoom. After reaching consensus, the wireframes were then used to program the actual mobile app on the Android OS platform using the Kotlin programming language. This step included determining the core underlying algorithm to classify the type of pain and recommend treatment options, based on the NAPCare PMG. Developing the algorithm was complex and done in consultation with pain experts within our team. Finally, we conducted multiple rounds of internal testing within our study team, as well as external pre-testing of the app with a small number of nurses/physicians in Nepal (n = 8) not directly involved with app design and development. A selection of screenshots of the current beta NAPCare PMG app 1.0 (with sample data) are displayed below (Figure 3) to provide a sense for the design and user interface of the app. The NAPCare PMG app is currently available on the Google Play Store as a privately available (not public) app to download for beta testing within our team. The key steps and timeline of our design process are summarized in Supplementary Table 1.

Importantly, design of the beta version of the app was informed by a comprehensive survey of oncology healthcare providers from four diverse cancer care institutions within Nepal (the same four sites where we later pilot tested the app). The goal of this survey was to better understand barriers and facilitators to cancer pain management and to gather end-user feedback about design of the mobile app (81). For example, a finding from our survey was that a key barrier to pain management was a high volume of patients. Therefore, we prioritized an app design that optimized drop-down menu choices that are quick and fast to use. Primary results from this survey that informed the design of the NAPCare PMG mobile app are presented in Table 3.

**Table 3:** Selected survey results that informed design of the mobile app.

**Key results from our survey of oncology healthcare providers at 4 cancer care centers in Nepal**	**How survey data informed design of the NAPCare PMG app**
*Knowledge, beliefs, and perceived barriers to cancer pain management* • 95% agree it is their job/role to manage cancer pain; • 95% agree that cancer pain can be difficult, but usually can be controlled; • Shortages of morphine, codeine and tramadol were rarely reported within the past 6 months at any institution; • Key barriers to pain management included lack of palliative care/pain management training for healthcare providers and having to care for too many patients; • Most common non-pharmacological treatments included: heat/cold packs and massage.	• Providers validate cancer pain management is an important patient care issue and opioids are usually available at their institution; • App user interface designed to be quick, simple and easy to use in busy clinical setting with multiple patient interactions happening simultaneously; • Enhanced app design will include more embedded tools related to opioid dosing, pain assessment strategies, side effect management, and links to palliative care training materials • App includes both pharmacological and non-pharmacological pain management strategies.
*Awareness and use of NAPCare PMG* • 96% had heard of the NAPCare PMG; 84% of nurses and 56% of physicians reported they have used the NAPCare PMG.	• The NAPCare PMG are a known and respected resource. Digitizing the NAPCare PMG has significant potential to increase uptake among larger numbers of generalist oncology HCPs.
*Smart phone usage and barriers* • 98% have access to a smart phone; 82% use an Android smart phone; • Apps are used ‘often' or ‘very often' by nurses and physicians for clinical care	• Smart phones are ubiquitous and commonly used by both nurses and physicians in Nepal and are a viable way to implement the PMG; • We built our app for Android OS, as it is most commonly used.
*Desired features of a pain mobile app* • Nurses: educate patients and family members; share information/learn from other healthcare providers; understand cancer pain physiology; • Physicians: help prescribe opioid medications; help prescribe non-opioid medications; share information with/learn from other healthcare providers.	• Enhanced app functionality will prioritize: (1) tools and resources to educate patients and family members; (2) support in safe opioid and non-opioid prescribing and administration; (3) understanding cancer pain physiology; and (4) sharing information with/learning from other healthcare providers.
*Outcome metrics/how we will know app is helping* • PMG will be followed more consistently; healthcare providers will feel more confident prescribing/administering pain management therapies; healthcare providers will use app often; families will be less stressed; patients will be in less pain.	• Outcome effectiveness measures for the app will include data tracking regarding frequency of app use; PMG adherence; HCP confidence in prescribing/administering pain therapies; patient reports of pain and perceived patient/family distress levels.

### Pilot Testing of the Mobile Application

#### Setting and Sample

After design of the mobile app, we conducted feasibility and acceptability testing of the NAPCare PMG mobile app with selected oncology healthcare providers using simulated patient case scenarios developed by the Nepal team. The final beta version of the app was deployed on Android study phones and a purposive quota sample of healthcare providers were recruited from four diverse study sites within Nepal (a public cancer hospital; a private cancer hospital; a public general hospital; and a hospice) to pilot test the app. These were the same 4 sites that also provided survey data that helped inform design of the app (81). The 4 study sites were carefully selected as each institution cares for adult patients with cancer and employed a key stakeholder of our study team affiliated with NAPCare, and also represented a diverse patient population and context. For example, private sector hospitals within Nepal generally care for a higher-income patient population and have enhanced resource availability, whereas public sector hospitals often care for patients of lower socioeconomic status with more constrained resources. Additionally, 3 of the sites were located within an urban setting, whereas the public cancer hospital was more rural.

Study participants were recruited primarily through word-of-mouth and screened for eligibility by a trained site study leader following a standardized protocol to conduct pilot testing in the field. We pre-loaded the app on Android study smartphones to ensure limited and controlled app distribution for beta testing. Study site leaders recruited healthcare providers aged 18 or over working in cancer or palliative care units who provide direct care to patients with cancer. The target number of participants for each study site was determined by the size of the institution (for example, the hospice facility operates with a small staff of approximately 10 healthcare providers and thus had a target of 7 providers), the scope of this pilot study, and what the Nepal research team members felt were realistic recruitment goals. The study was approved by respective ethical committees (UVA Social and Behavioral Sciences Institutional Review Board and the Nepal Health Research Council), prior to any data collection.

After providing informed consent, basic demographic data were collected and participants were provided a study smartphone preloaded with the NAPCare PMG app. Participants were then asked to use the app to work through two (out of four) randomly selected representative cancer pain patient case studies, written by members of the Nepal research team (Supplementary Table 2). These case studies represented various types of common pain scenarios seen by clinicians in Nepal. Data had the ability to be entered off-line (i.e., without internet connectivity), stored, and then later pushed to a secure automation tool (Integromat) when the user reconnected to the internet. Integromat then created a row in a Google spreadsheet to store app response data.

After participants finished using the app, they completed a brief Qualtrics survey about their experience on a study tablet. This survey included the industry-standard 10-item System Usability Scale (83, 84) (SUS) and selected items (*n* = 12) from the Mobile App Rating Scale (85–87) (MARS) to assess functionality, aesthetics, information, subjective quality, and perceived impact. An open text field for additional comments or suggestions was also included. The survey was designed in consultation with the UVA Center for Survey Research and reviewed by a native Nepali speaker not directly involved in the study; some survey items were modified slightly for clarity. Study tablets and phones had data plans added to reduce the risk of problems with potentially unstable institution WiFi.

#### Data Analysis

Qualtrics survey data were cleaned and exported for analysis. Descriptive and summary statistics were completed and compared across institutions and roles, and open-text responses compiled and summarized. The SUS was calculated as recommended, with a range of scores from 0 to 100, with a score of 68 considered above average (88). The MARS overall score was calculated as a simple average of the 12 items from the survey, and we also calculated subgroup MARS category scores (function; aesthetics; information; subjective quality, perceived impact). Additionally, we conducted regression analyses to explore relationships between selected demographic variables and SUS and MARS scores. Specifically, overall SUS and MARS scores were modeled using the Generalized Linear Model (GLM) procedure and variables considered included age, clinical role, institution, primary practice area, total years as a nurse or physician, formal training in palliative care, and pre-existing familiarity with the NAPCare PMG. These variables were selected as we hypothesized they may be most influential in predicting how a respondent experienced the NAPCare PMG app. To identify covariates with potential significance in modeling overall SUS and MARS, univariate models of SUS and MARS were initially considered. Only variables with a *p*-value <0.10 were considered for the final model of SUS and MARS. In order to establish the final model for these outcomes, all variables significant in univariate models were combined. Covariates were then sequentially removed by highest *p*-value if no longer significant in the presence of other covariates at α = 0.05 level. All analyses were performed using SPSS 27.0 (IBM SPSS, Chicago, IL) and SAS 9.4 (SAS Institute, Cary, NC). Due to small group sizes, several variables were regrouped, combining similar subgroups for use in the models. These variables included: clinical role, combining “MD” and “MD student”; combining providers aged 41 and older; condensing primary practice area groups to “Medical oncology,” “Palliative care” and “Other”; and combining total years as a nurse or physician to responses of 5 years or less in a single group.

## Results

### Participant Demographics

A total of 98 healthcare providers (*n* = 98) across four cancer care centers within Nepal pilot tested the NAPCare PMG app (Table 4). The majority of participants were nurses (*n* = 70; 71.4%) with 22 physicians (*n* = 22; 22.4%) and 6 medical students (*n* = 6; 6.1%). Most participants were female (*n* = 79; 80.6%), had completed a Bachelor's degree (*n* = 50; 51%) and almost half of respondents were between ages 18–30 (*n* = 47; 48%). An equal number of respondents were from the public cancer hospital, private cancer hospital and public general hospital (*n* = 30; 30.6%) and a smaller number from the hospice, which generally only has a total staff of about 10 people (*n* = 8; 8.2%). An almost equal number of participants reported completing formal training in palliative care/cancer pain management (*n* = 48; 49.0%) versus not (*n* = 50; 51.0%). Almost half of participants (*n* = 47; 48%) reported being “very familiar” with the NAPCare PMG (score of 8 or higher on a 0, no familiarity to 10, very familiar, scale); 28.6% (*n* = 28) participants reported they “never” use mobile apps to provide clinical care to patients, whereas 21.4% (*n* = 21) reported using mobile apps for clinical care “very often” (score of 8 or higher on 0, never to 10, very often). Many more participants (*n* = 64; 65.3%) reported using mobile apps for personal reasons “very often” (score of 8 or higher on 0, never to 10, very often); only 5 participants (*n* = 5; 5.1%) said they “never” use mobile apps for personal reasons.

**Table 4:** Demographics of participants who tested the NAPCare PMG mobile application.

	**Institution**								**All**	
	**Public hospital, general**		**Public hospital, cancer**		**Hospice**		**Private hospital, cancer**			
	***N***	**%**	***N***	**%**	***N***	**%**	***N***	**%**	***N***	**%**
**Age**										
18–30 years old	17	56.7	4	13.3	5	62.5	2	70.0	4	48.
31–40 years old	9	30.0	10	33.3	1	12.5	9	30.0	29	29.6
41–50 years old	3	10.0	15	50.0	2	25.0	0	0.0	20	20.4
51–60 years old	1	3.3	1	3.3	0	0.0	0	0.0	2	2.0
**Clinical role**										
Nurse	21	70.0	20	66.7	7	87.5	22	73.3	70	71.4
Physician	3	10.0	10	33.3	1	12.5	8	26.7	22	22.4
Medical student	6	20.0	0	0.0	0	0.0	0	0.0	6	6.1
**Institution**										
Public hospital, general	30	100.0	0	0	0	0	0	0	30	30.6
Public hospital, cancer	0	0	30	100.0	0	0	0	0	30	30.6
Hospice	0	0	0	0	8	100.0	0	0	8	8.2
Private hospital, cancer	0	0	0	0	0	0	30	100.0	30	30.6
**Gender**										
Male	6	20.0	10	33.3	0	0.0	3	10.0	19	19.4
Female	24	80.0	20	66.7	8	100.0	27	90.0	79	80.6
**Highest completed level of education**										
PCL (Proficiency Certificate Level)	8	26.7	2	6.7	4	50.0	10	33.3	24	24.5
Bachelors	17	56.7	14	46.7	2	25.0	17	56.7	50	51.0
Post-Graduate	5	16.7	14	46.7	2	25.0	3	10.0	24	24.5
**Primary current practice area**										
Medical oncology	26	86.7	3	10.0	1	12.5	20	66.7	50	51.0
Surgical oncology	0	0.0	10	33.3	0	0.0	1	3.3	11	11.2
Radiation oncology	3	10.0	3	10.0	0	0.0	0	0.0	6	6.1
Palliative care	0	0.0	12	40.0	7	87.5	9	30.0	28	28.6
General ward (in-patient)	1	3.3	0	0.0	0	0.0	0	0.0	1	1.0
Other (anesthesia)	0	0.0	2	6.7	0	0.0	0	0.0	2	2.0
**Years total as a nurse or physician**										
Less than 1 year	1	3.3	0	0.0	2	25.0	0	0.0	3	3.1
1–5 years	13	43.3	1	3.3	4	50.0	22	73.3	40	40.8
6–10 years	8	26.7	5	16.7	0	0.0	6	20.0	19	19.4
More than 10 years	7	23.3	24	80.0	2	25.0	2	6.7	35	35.7
Does not apply; I am a trainee/student	1	3.3	0	0.0	0	0.0	0	0.0	1	1.0
**Have you completed formal training in palliative care and/or cancer pain management?**										
Yes	14	46.7	17	56.7	8	100.0	9	30.0	48	49.0
No	16	53.3	13	43.3	0	0.0	21	70.0	50	51.0
**Which types of training have you completed? (select all that apply)** ***Asked of those who answered “yes” to completing formal training***										
Workshops/classes (1–3 days)	5	16.7	6	20.0	1	12.5	2	6.7	14	14.3
Training courses (weeks to months)	9	30.0	10	33.3	5	62.5	6	20.0	30	30.6
Certificate programs (months to years)	0	0.0	1	3.3	0	0.0	1	3.3	2	2.0
Fellowships (months to years)	0	0.0	0	0.0	2	25.0	0	0.0	2	2.0

### Descriptive Survey Results

**Figure 4:**
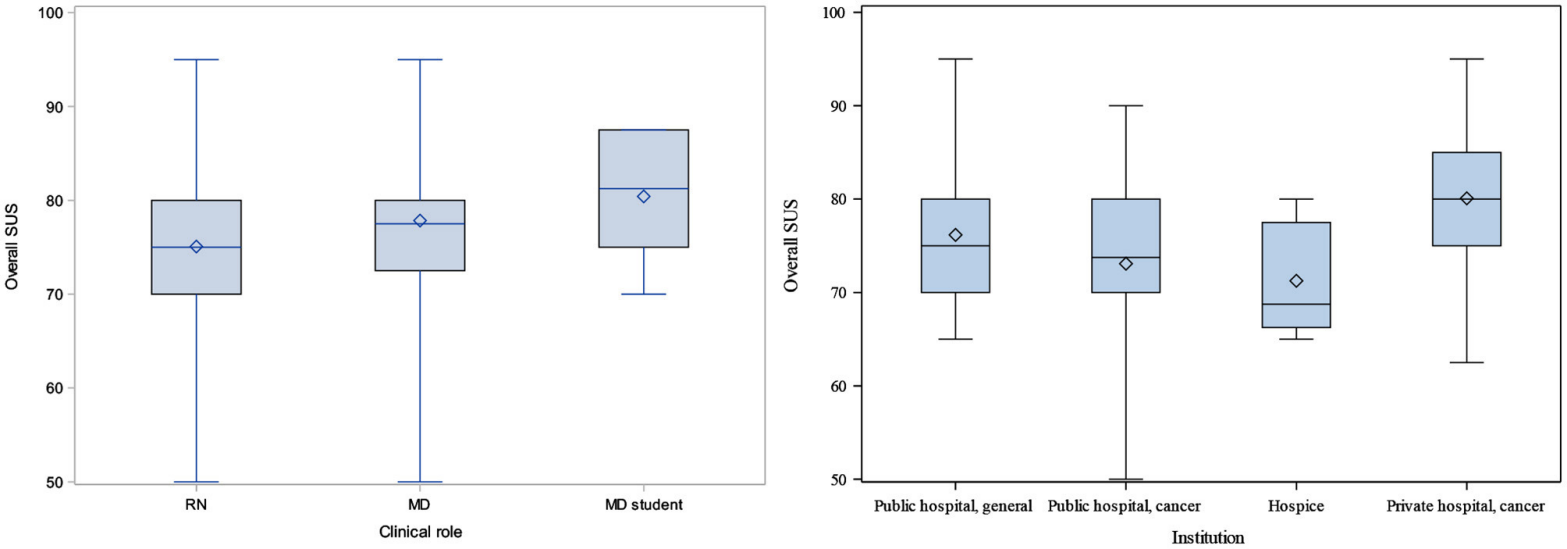
Overall SUS scores by clinical role (left) and institution (right).

**Figure 5:**
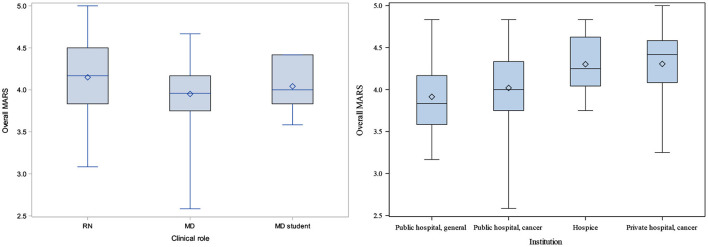
Overall MARS scores by clinical role (L) and institution (R).

Overall, across institutions and roles, the app received an SUS score of 76.0 (a score above 68 is considered above average). Table 5 displays individual SUS survey items and responses across institutions, with preferred responses in italics. The overall MARS average, across institutions and roles, was 4.10 (on a scale of 1 = poor, 5 = excellent). Over 75% of the app testers (*n* = 75; 76.1%) rated the app as 4 or 5 stars (out of 5); 85% (*n* = 83) “agreed” or “strongly agreed” that the app increased their awareness of the importance of managing cancer pain; 86% (*n* = 84) “agreed” or “strongly agreed” that the app increased their knowledge of managing cancer pain; and 89.8% (*n* = 88) “agreed” or “strongly agreed” that the app will help them better manage cancer pain. Over 60% (*n* = 61; 62.2%) reported they would recommend the NAPCare PMG to all nurses and physicians. Table 6 displays MARS survey items and responses across institutions; Table 7 displays MARS responses, grouped by subcategories, across institutions. Summary SUS and MARS results are displayed in Figures 4, 5 and Table 8. A total of 44 free-text responses were recorded (32 from nurses; 12 from physicians/medical students); the majority provided feedback regarding specific medications and dosing recommendations.

**Table 5:** SUS survey responses, by institution.

SUS survey Item^*^	**Institution**								**All**	
	**Public hospital, general**		**Public hospital, cancer**		**Hospice**		**Private hospital, cancer**		
	***N***	**%**	***N***	**%**	***N***	**%**	***N***	**%**	***N***	**%**
**I would like to use this app frequently (SUS 1)**										
*Strongly agree*	*11*	*36.7*	*12*	*40.0*	*0*	*0.0*	*9*	*30.0*	*32*	*32.7*
*Agree*	*19*	*63.3*	*17*	*56.7*	*8*	*100.0*	*21*	*70.0*	*65*	*66.3*
Neither agree nor disagree	0	0.0	1	3.3	0	0.0	0	0.0	1	1.0
**This app is too complicated. (SUS 2)**										
Strongly agree	0	0.0	1	3.3	0	0.0	0	0.0	1	1.0
Agree	0	0.0	4	13.3	0	0.0	0	0.0	4	4.1
Neither agree nor disagree	0	0.0	1	3.3	0	0.0	0	0.0	1	1.0
*Disagree*	*28*	*93.3*	*20*	*66.7*	*8*	*100.0*	*27*	*90.0*	*83*	*84.7*
*Strongly disagree*	*2*	*6.7*	*4*	*13.3*	*0*	*0.0*	*3*	*10.0*	*9*	*9.2*
**This app is easy to use (SUS 3)**										
*Strongly agree*	*5*	*16.7*	*10*	*33.3*	*1*	*12.5*	*13*	*43.3*	*29*	*29.6*
*Agree*	*23*	*76.7*	*17*	*56.7*	*7*	*87.5*	*17*	*56.7*	*64*	*65.3*
Neither agree nor disagree	2	6.7	2	6.7	0	0.0	0	0.0	4	4.1
Disagree	0	0.0	1	3.3	0	0.0	0	0.0	1	1.0
**I need help from a technical person to use this app (SUS 4)**										
Agree	4	13.3	6	20.0	2	25.0	2	6.7	14	14.3
Neither agree nor disagree	0	0.0	2	6.7	3	37.5	2	6.7	7	7.1
*Disagree*	*18*	*60.0*	*18*	*60.0*	*3*	*37.5*	*19*	*63.3*	*58*	*59.2*
*Strongly disagree*	*8*	*26.7*	*4*	*13.3*	*0*	*0.0*	*7*	*23.3*	*19*	*19.4*
**The functions of this app are logical/make sense to me (SUS 5)**										
*Strongly agree*	*4*	*13.3*	*9*	*30.0*	*1*	*12.5*	*10*	*33.3*	*24*	*24.5*
*Agree*	*24*	*80.0*	*21*	*70.0*	*7*	*87.5*	*19*	*63.3*	*71*	*72.4*
Neither agree nor disagree	2	6.7	0	0.0	0	0.0	1	3.3	3	3.1
**This app is inconsistent (SUS 6)**										
Agree	0	0.0	4	13.3	0	0.0	1	3.3	5	5.1
Neither agree nor disagree	1	3.3	2	6.7	0	0.0	2	6.7	5	5.1
*Disagree*	*24*	*80.0*	*21*	*70.0*	*7*	*87.5*	*23*	*76.7*	*75*	*76.5*
*Strongly disagree*	*5*	*16.7*	*3*	*10.0*	*1*	*12.5*	*4*	*13.3*	*13*	*13.3*
**Most people could learn to use this app quickly (SUS 7)**										
*Strongly agree*	*2*	*6.7*	*6*	*20.0*	*1*	*12.5*	*11*	*36.7*	*20*	*20.4*
*Agree*	*28*	*93.3*	*22*	*73.3*	*7*	*87.5*	*19*	*63.3*	*76*	*77.6*
Neither agree nor disagree	0	0.0	1	3.3	0	0.0	0	0.0	1	1.0
Disagree	0	0.0	1	3.3	0	0.0	0	0.0	1	1.0
**This app is very awkward to use (SUS 8)**										
Agree	1	3.3	6	20.0	0	0.0	0	0.0	7	7.1
Neither agree nor disagree	0	0.0	1	3.3	0	0.0	1	3.3	2	2.0
*Disagree*	*23*	*76.7*	*23*	*76.7*	*6*	*75.0*	*22*	*73.3*	*74*	*75.5*
*Strongly disagree*	*6*	*20.0*	*0*	*0.0*	*2*	*25.0*	*7*	*23.3*	*15*	*15.3*
**I felt very confident using this app (SUS 9)**										
*Strongly agree*	*4*	*13.3*	*5*	*16.7*	*1*	*12.5*	*11*	*36.7*	*21*	*21.4*
*Agree*	*24*	*80.0*	*25*	*83.3*	*7*	*87.5*	*18*	*60.0*	*74*	*75.5*
Neither agree nor disagree	2	6.7	0	0.0	0	0.0	1	3.3	3	3.1
**I needed to learn a lot of things before I could use this app (SUS 10)**										
Strongly agree	0	0.0	1	3.3	1	12.5	0	0.0	2	2.0
Agree	7	23.3	7	23.3	4	50.0	2	6.7	20	20.4
Neither agree nor disagree	5	16.7	3	10.0	1	12.5	1	3.3	10	10.2
*Disagree*	*15*	*50.0*	*18*	*60.0*	*2*	*25.0*	*23*	*76.7*	*58*	*59.2*
*Strongly disagree*	*3*	*10.0*	*1*	*3.3*	*0*	*0.0*	*4*	*13.3*	*8*	*8.2*

* *If response option is not displayed, no participant selected this option. Preferred survey responses are noted in italics*.

**Table 6:** MARS survey responses, by institution.

MARS survey Item^*^	**Institution**								**All**	
	**Public hospital, general**		**Public hospital, cancer**		**Hospice**		**Private hospital, cancer**		
	***N***	**%**	***N***	**%**	***N***	**%**	***N***	**%**	***N***	**%**
**Function**										
**How well does the app work?**										
Some functions work, but there are major technical problems.	0	0.0	1	3.3	0	0.0	0	0.0	1	1.0
App works overall. Some minor technical problems. Slow at times.	5	16.7	1	3.3	1	12.5	2	6.7	9	9.2
Mostly functions well without problems.	21	70.0	22	73.3	4	50.0	16	53.3	63	64.3
Perfect, prompt response. No technical problems.	4	13.3	6	20.0	3	37.5	12	40.0	25	25.5
**How easy is it to learn how to use the app?**										
A little difficult	6	20.0	3	10.0	0	0.0	2	6.7	11	11.2
Easy	21	70.0	24	80.0	7	87.5	19	63.3	71	72.4
Very easy	3	10.0	3	10.0	1	12.5	9	30.0	16	16.3
**Does moving between the screens make sense?**										
No. It is confusing.	0	0.0	2	6.7	0	0.0	0	0.0	2	2.0
Understandable after much time/effort.	2	6.7	1	3.3	1	12.5	0	0.0	4	4.1
Understandable after some time/effort.	3	10.0	3	10.0	0	0.0	3	10.0	9	9.2
Easy to understand with practice.	25	83.3	23	76.7	7	87.5	19	63.3	74	75.5
Very logical and intuitive screen flow.	0	0.0	1	3.3	0	0.0	8	26.7	9	9.2
**Aesthetics**										
**How clear is arrangement of buttons, menus, and content on the screen?**										
Satisfactory.	15	50.0	12	40.0	3	37.5	9	30.0	39	39.8
Mostly clear. Able to select, locate, see, read items.	13	43.3	11	36.7	4	50.0	7	23.3	35	35.7
Very clear and simple.	2	6.7	7	23.3	1	12.5	14	46.7	24	24.5
**How does the app look, in general?**										
OK. Average looking.	16	53.3	10	33.3	1	12.5	5	16.7	32	32.7
Pretty good. Nice to look at.	10	33.3	14	46.7	4	50.0	10	33.3	38	38.8
Beautiful. Very professional.	4	13.3	6	20.0	3	37.5	15	50.0	28	28.6
**Information**										
**How accurate/correct is the information in the app?**										
A little accurate/correct. There are some errors.	2	6.7	1	3.3	0	0.0	1	3.3	4	4.1
I am not sure if there are any errors.	10	33.3	10	33.3	0	0.0	2	6.7	22	22.4
Mostly accurate/correct. There are a few errors.	10	33.3	8	26.7	5	62.5	9	30.0	32	32.7
Very accurate/correct. There are no errors.	8	26.7	11	36.7	3	37.5	18	60.0	40	40.8
**Subjective quality**										
**Would you recommend this app to other nurses and/or physicians?**										
No. I would not recommend this app to other nurses or physicians.	1	3.3	0	0.0	0	0.0	0	0.0	1	1.0
There are very few nurses/physicians I would recommend this app to.	0	0.0	2	6.7	0	0.0	1	3.3	3	3.1
Maybe; there are several nurses/physicians I would recommend this app to.	0	0.0	5	16.7	0	0.0	2	6.7	7	7.1
There are many nurses/physicians I would recommend this app to.	13	43.3	8	26.7	2	25.0	3	10.0	26	26.5
Yes, definitely. I would recommend this app to all nurses/physicians.	16	53.3	15	50.0	6	75.0	24	80.0	61	62.2
**How many times would you use this app in the next year?**										
1–2 times	2	6.7	3	10.0	0	0.0	0	0.0	5	5.1
3–10 times	3	10.0	6	20.0	0	0.0	1	3.3	10	10.2
11–50 times	5	16.7	10	33.3	1	12.5	14	46.7	30	30.6
>50 times	20	66.7	11	36.7	7	87.5	15	50.0	53	54.1
**What is your overall (star) rating of this app?**										
3 stars; average	18	60.0	5	16.7	0	0.0	0	0.0	23	23.5
4 stars	8	26.7	20	66.7	6	75.0	22	73.3	56	57.1
5 stars; one of the best apps I've used	4	13.3	5	16.7	2	25.0	8	26.7	19	19.4
**Perceived impact**										
**This app increased my awareness of the importance of managing cancer pain**.										
Strongly disagree	0	0.0	0	0.0	0	0.0	1	3.3	1	1.0
Disagree	1	3.3	0	0.0	0	0.0	1	3.3	2	2.0
Neither agree nor disagree	9	30.0	2	6.7	0	0.0	1	3.3	12	12.2
Agree	12	40.0	21	70.0	3	37.5	17	56.7	53	54.1
Strongly agree	8	26.7	7	23.3	5	62.5	10	33.3	30	30.6
**This app increased my knowledge of managing cancer pain**.										
Neither agree nor disagree	9	30.0	2	6.7	0	0.0	3	10.0	14	14.3
Agree	14	46.7	19	63.3	5	62.5	19	63.3	57	58.2
Strongly agree	7	23.3	9	30.0	3	37.5	8	26.7	27	27.6
**This app will help me better manage cancer pain**.										
Neither agree nor disagree	5	16.7	2	6.7	0	0.0	3	10.0	10	10.2
Agree	16	53.3	19	63.3	6	75.0	16	53.3	57	58.2
Strongly agree	9	30.0	9	30.0	2	25.0	11	36.7	31	31.6

* *If response option is not displayed, no participant selected that particular option*.

**Table 7:** Summary of MARS scores, by subcategories.

	**Institution**												**All**		
	**Public hospital, general**			**Public hospital, cancer**			**Hospice**			**Private hospital, cancer**			
	***N***	**Mean**	**St Dev**	***N***	**Mean**	**St Dev**	***N***	**Mean**	**St Dev**	***N***	**Mean**	**St Dev**	***N***	**Mean**	**St Dev**
**Overall MARS**	30	3.91	0.41	30	4.02	0.46	8	4.30	0.38	30	**4.31**	0.44	98	**4.10**	0.46
MARS: function	30	3.88	0.43	30	3.92	0.50	8	4.04	0.49	30	**4.24**	0.45	98	**4.02**	0.48
MARS: aesthetics	30	3.58	0.54	30	3.85	0.68	8	4.00	0.65	30	**4.25**	0.70	98	**3.90**	0.69
MARS: information	30	3.80	0.92	30	3.97	0.93	8	4.38	0.52	30	**4.47**	0.78	98	**4.10**	0.89
MARS: subjective quality	30	4.13	0.48	30	4.06	0.67	8	**4.63**	0.28	30	4.47	0.46	98	**4.25**	0.56
MARS: perceived impact	30	3.99	0.68	30	4.21	0.52	8	**4.42**	0.43	30	4.19	0.66	98	**4.15**	0.62

*The bold values indicate the highest scores*.

**Table 8:** Overall SUS and MARS scores, by institution and role.

	**Overall SUS**			**Overall MARS**		
	***N***	**Mean**	**St Dev**	***N***	**Mean**	**St Dev**
**Institution**
Public hospital, general	30	76.17	7.45	30	3.91	0.41
Public hospital, cancer	30	73.08	10.12	30	4.02	0.46
Hospice	8	71.25	6.27	8	4.30	0.38
Private hospital, cancer	30	**80.08**	8.21	30	**4.31**	0.44
**Clinical role**
RN	70	75.07	8.74	70	**4.15**	0.46
MD	22	77.84	9.71	22	3.95	0.49
MD student/trainee	6	**80.42**	6.97	6	4.04	0.34
All	98	**76.02**	8.94	98	**4.10**	0.46

*The bold values indicate the highest scores*.

### Modeling Results

**Figure 6:**
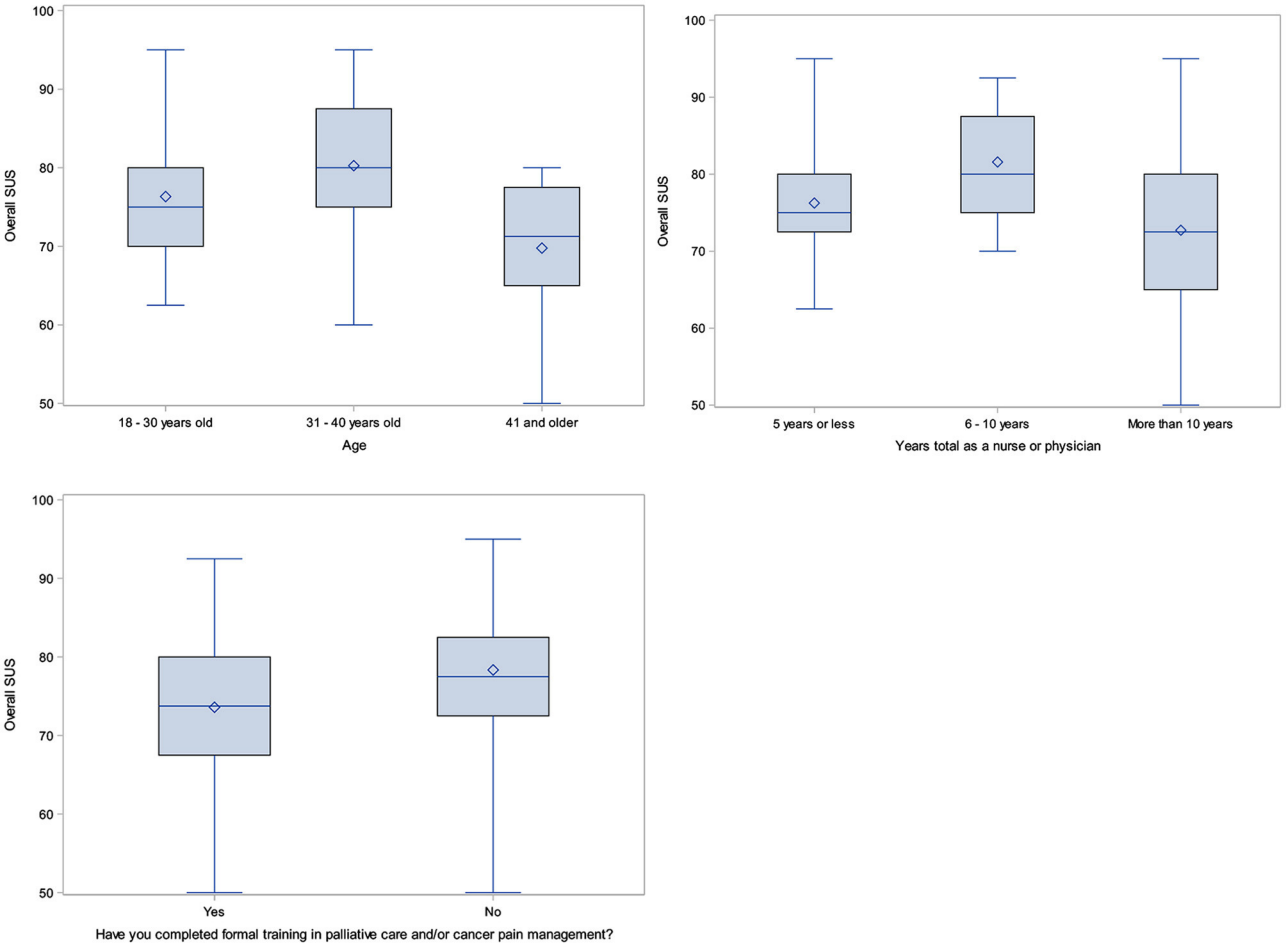
Boxplots of statistically significant variables in final multivariate SUS models.

Nine variables were considered in modeling overall SUS scores (Supplementary Table 3). Seven factors were significant in univariate models: age, clinical role, institution, total years as a nurse or physician, formal training in palliative care, use of mobile apps for clinical care, and use of mobile apps for personal use. Once combined in a multivariate model, only age, years of experience, and training in palliative care were significant in modeling SUS (*p*-values, 0.0124, 0.0371, and 0.0189, respectively). Supplementary Table 4 indicates that participants under 41 years old have higher SUS than those who are 41 and older, having 6 to 10 years of experience is associated with the highest estimates of SUS, and having no formal training in palliative care is associated with higher SUS. Boxplots (Figure 6) display the relationships between SUS and variables significant in the SUS model.

Among 9 demographic variables considered in the initial MARS modeling, 2 were significant in univariate models (Supplementary Table 5): clinical role and institution (*p*-values 0.0834 and 0.0030, respectively). Once combined in a multivariate model (Supplementary Table 6), only institution remained significant in modeling MARS (*p*-value = 0.0030). Hospice and the private cancer hospital were associated with the highest MARS scores; the general public hospital was associated with the lowest MARS scores. Boxplots of overall MARS by institution display the differences in MARS across institution (Figure 5).

## Discussion

### Overall Results and the App Design Process

Overall, users reported very positive feedback and receptivity regarding the NAPCare PMG mobile app. Our results demonstrate strong feasibility and acceptability of oncology care providers in Nepal using the NAPCare PMG mobile app to help guide cancer pain care. Across diverse care contexts, and care provider roles, the overall SUS (72/100) and MARS scores (4.1/5) indicate users find the app useful, easy to navigate, and helpful. Potential uptake of the app is further supported by our findings that smartphone use is ubiquitious and that healthcare providers commonly use mobile apps both for clinical and personal reasons. It is also encouraging that the majority of open-text feedback and suggestions related to the app focused on very specific—and highly fixable—medication and dosing changes, vs. larger structural or conceptual concerns. Our research adds to the existing and rapidly expanding body of literature related to the use of mobile apps in LMICs to support healthcare providers and patient care (41, 89), the benefits of global partnerships to strengthen research capacity (90–92), and the value of using a community based participatory approach in designing mobile apps (73, 79). Importantly, our findings contribute knowledge related to the importance of developing health-related mobile apps that are congruent with the local cultural and resource context. We see a key strength of this work as leveraging the pre-existing NAPCare PMG which were created by community and clinical leaders within Nepal and already had a high degree of buy-in from healthcare providers prior to app development. We also find it noteworthy that we were able to accomplish the aims of this grant despite the severe disruption of the COVID-19 global pandemic, which disportionately impacted our Nepal team members. That we were able to successfully complete development and pilot testing of the app despite the myriad and unpredictable challenges wrought by COVID-19 is a testament to the dedication, commitment and tenacity of our partners in Nepal.

In terms of our app development design process, our in-person workshop in January 2020 was extremely productive; this proved very fortitious as future planned in-person workshops were impossible due to COVID-19 travel restrictions. The remaining app design work subsequently occurred over Zoom, which had its challenges, but was made significantly easier by the foundational work we had successfully completed during the prior in-person workshop. The January workshop was particularly effective due to the hands-on and interactive aspect of the work, which was assisted by printing out the large posters which visually represented the app flow. Working through potential case studies using the poster, and identifying problems, was one of the most effective activities during our workshop and proved invaluable in determining the finalized app algorithm. Our project also benefitted from the engagement of a highly collaborative software engineer who deftly navigated cross-cultural communications and interactions. It is important to note that while mobile app development and pilot testing was a key deliverable of this project, the overarching and larger goal was building research capacity and solidifying our collaboration.

### MARS and SUS Results

We used a combination of descriptive and inferential statistical analysis to more fully understand the user experience related to pilot testing the NAPCare PMG and to help inform future work. Importantly, our regression analyses suggests end-user variables important to consider (age, years of experience, training in palliative care, and institutional setting) in designing and evaluating mobile apps to improve cancer pain management in lower resourced settings.

#### Descriptive

By institution, both the overall average SUS and MARS scores were highest for the private cancer hospital (80.1; 4.31, respectively). By role, the SUS scores were highest for medical students (80.4) and MARS scores were highest for nurses (4.15). One possible explanation for the higher SUS and MARS scores in the private cancer hospital is that this group of providers, given their work in a generally higher-resourced context, may have had more exposure to in-services and palliative care training, and perhaps more interest and experience in using apps to guide patient care. Interpreting SUS and MARS results by role is perhaps less straightforward and should be done cautiously as we had a small group of medical students (*n* = 6) and, with MARS, smaller differences between provider roles.

An additional challenge is knowing how to best interpret divergent results between overall SUS with MARS scores. For example, hospice had the lowest overall SUS scores (71.3), but the second highest MARS score (4.30, just behind the private cancer hospital at 4.31). Whether this is more reflective of the SUS and MARS capturing different facets of the user experience, or truly represents a difference between institutional mobile app needs, requires further exploration—as was done with our modeling analysis (see below). One noteworthy finding from our prior survey (81) related to differences in permissibility of the use of mobile phones in the clinical work setting between physicians and nurses (nurses reported being less likely to be allowed to use mobile phones in the work setting). As our hospice participants were predominantly nurses (*n* = 7, 88%), perhaps this impacted their experience testing the app and SUS scores.

MARS average scores by subcategories (function, aesthetics, information, subjective quality, and perceived impact) allows for a more granular exploration of why results may differ across institutions. Subjective quality and perceived impact were rated most highly by hospice (4.63, 4.42, respectively), whereas function, aesthetics and information were rated most highly by the private cancer hospital (4.24, 4.25, 4.47, respectively). Higher scores in these areas by hospice may be because nurses in this practice environment frequently need to verify information using Google or reference books in the hospice library. The NAPCare PMG app may be seen as particularly impactful in this type of care setting as it reduces the need for additional verification and provides reassurance related to the accuracy and quality of the information. Additionally, hospice had the highest percentage of participants younger than 30 years of age—individuals who are potentially more comfortable and receptive to mobile apps—which may have contributed to higher MARS scores. Higher scores by the private cancer hospital may be related to the generally higher resource context of private sector hospitals, and also that the private cancer hospital included in our study has a separate palliative care ward and specific dedicated palliative care nurses. Conversely, the lower MARS scores reported by the public general hospital may be due to the lack of participants who worked specifically in palliative care.

#### Modeling

Our final multivariate models revealed that age (<41 years), years in practice (6–10 years), and previous training in palliative care/cancer pain management (no) were all significant in predicting higher SUS scores. While younger age and more years in practice seem logical in predicting higher SUS scores (e.g., younger participants may have more familiarity/ease in using mobile apps and healthcare providers with a fair amount of clinical experience recognize the value of the app to help manage cancer pain), the finding related to previous training is interesting. One possible explanation is that healthcare providers with previous formal training in palliative care or cancer pain management, and a greater fund of knowledge in these areas, expected *more* from the app, and were more attuned to areas for improvement, or corrections needed with specific pharmacological recommendations. Therefore, providers without palliative care or cancer pain management training may have been less critical of the app, resulting in higher SUS scores for this participant group.

The only significant variable in predicting MARS scores was institution, with hospice and the private cancer hospital associated with higher MARS scores, and the public cancer hospital associated with lower MARS scores (In our descriptive analysis, the private cancer hospital also had the highest overall average SUS and MARS scores.). This finding supports our initial hypothesis that mobile app needs and preferences will vary across different clinical contexts. Possible reasons for these differences across institutions may relate to the volume of cancer patients seen, presence of dedicated palliative care beds/wards and specialized palliative care personnel, and staffing turnover, which may affect knowledge and attitudes regarding pain management and palliative care.

With our modeling analysis, differing variables of significance between the SUS and MARS are most likely attributed to the fact that these tools are evaluating different facets of the user experience, and thus different aspects are important in predicting scores. For example, the SUS focuses more on the user experience with technology systems in general, while the MARS is more specifically designed to assess the user experience with mobile apps. This finding also highlights the challenges of measurement (in general, but in this case, specifically in evaluating technology) across cultures and of ensuring a shared understanding of what different tools are actually measuring, especially in diverse populations and healthcare contexts. Interestingly, “clinical role” as a demographic variable was not found to be significant with either the SUS or MARS, although this warrants further exploration in future work as our descriptive findings and prior work suggests there may be important disciplinary considerations in the use of mobile apps to support patient care.

### Limitations

The primary limitation of this study is the relatively small sample size of palliative care-sensitized healthcare providers. However, our sampling strategy and size is consistent with the scale and scope of a pilot feasibility and acceptability study (93–95), and must be considered in the context of a global project conducted during the COVID-19 pandemic. Additionally, most of our participants were nurses, as at all sites more nurses are employed than physicians. We beta tested the app using simulated case studies vs. clinical encounters with real patients; again, this approach is consistent with a pilot study designed to assess user experience of the app vs. clinical effectiveness. One particular challenge of this work related to finding the best tool(s) to evaluate the mobile app. After extensive searching in the literature, we ultimately decided on the SUS and selected items from the MARS. However, both tools required some adapation in terms of simplifying and clarifying language with input from our Nepal team members and an external native Nepali speaker/interpreter consultant. An important area of future work should be the development of concise, simple tools to meaningfully evaluate mobile apps in lower-resourced settings with diverse user groups.

## Conclusion

Overall, and across institutions and roles, the NAPCare PMG mobile app was extremely well-received, and participants rated it highly on both the SUS and MARS. Regression analyses suggest end-user variables important to consider (e.g., age, years of experience, training in palliative care, and institutional setting) in designing and evaluating mobile apps in lower resourced settings. Healthcare providers within LMICs can utilize mobile apps to improve cancer pain care and support adherence to clinical practice guidelines, but it is critical these tools are culturally and contextually congruent. Our app design and pilot testing process illustrate the benefits of cross global collaborations to build research capacity and generate knowledge within the local context. Using locally developed PMG—vs. those imported from the West—is a key strength of this work and offers a scalable approach that can be reproduced in other LMICs. Future work will include enhancing features and functionality of the NAPCare PMG app and testing its efficacy in real-world clinical encounters on relevant healthcare provider, patient, and organizational outcomes with a larger and more diverse sample of participants.

## Data Availability Statement

De-identified data, in compliance with institutional data sharing requirements, will be made available by the authors upon reasonable request.

## Ethics Statement

This study was reviewed and approved by University of Virginia Social and Behavioral IRB and the Nepal Health Research Council. The participants provided their informed consent to participate in this study as per IRB guidelines. Written informed consent was obtained from the relevant individuals, for the publication of any potentially identifiable images or data included in this article.

## Author Contributions

VL, AA, SC, RG, RK, GK, MM, DM, BN, KS, RS, SS, UT, RD, and BP conceptualized the study. BHa wrote the software code for the mobile app. VL, AA, SC, MD, RG, BHa, RK, GK, MM, DM, BN, KS, RS, AS, SS, UT, and BP conducted and/or supported data collection. VL, BHa, and AK led data analysis. VL, BHa, SC, GK, AK, MM, RS, UT, RD, and BP assisted with interpretation of findings. VL wrote first draft of manuscript and incorporated feedback from co-authors into the final paper. All co-authors reviewed and approved the final manuscript.

## Funding

This research was supported by the Fogarty International Center, National Institutes of Health, R21TW011244.

## Conflict of Interest

BHa is the founder of and employed by Hass Software Consulting. RD provides consultative services for Warm Health Technologies, Inc. The remaining authors declare that the research was conducted in the absence of any commercial or financial relationships that could be construed as a potential conflict of interest.

## Publisher's Note

All claims expressed in this article are solely those of the authors and do not necessarily represent those of their affiliated organizations, or those of the publisher, the editors and the reviewers. Any product that may be evaluated in this article, or claim that may be made by its manufacturer, is not guaranteed or endorsed by the publisher.
