# Supplementary material for: A Global Collaboration to Develop and Pilot Test a Mobile Application to Improve Cancer Pain Management in Nepal
Source: Front Pain Res (Lausanne). 2022 Jul 28;3:910995. doi: 10.3389/fpain.2022.910995 (PMC9366104; doi:10.3389/fpain.2022.910995)
Supplement: Supplementary file 1 [file Data_Sheet_1.pdf]

Supplementary Materials

Supplementary Table 1. Key steps and timeline in our mobile app design and beta testing process.

November – December 2019

- Engineering team provided with paper version of the NAPCare PMG; discussed with team investigators to understand clinical context of project.
- Engineering team mapped the NAPCare PMG to decision flowcharts and created first drafts of wireframes and high-fidelity app-screen mock-ups; shared with study team to gather preliminary feedback.

January 2020

A 2-day workshop was held in Nepal with study team members. Key activities included:

- Presented updated decision flowcharts, wireframes and high-fidelity mock-ups and solicited additional feedback from team members.
- Reached consensus regarding 'need', 'nice' and 'next' preferences for the app.
- Critique of decision flowcharts using large, vinyl posters and case study examples.

February 2020 – January 2021

- Finalized app decision flowcharts and high-fidelity mock-ups.
- Determined underlying pain decision algorithm.
- Code for app written.
- Conducted internal and pre-testing of app and incorporated feedback.

February 2021

- Pilot testing conducted at the four study sites within Nepal.

Supplementary Table 2. Exemplar patient case studies developed by the Nepal team to pilot test the app.

Case Study 1
Mr. Ram Lama 60 years male diagnosed patient of lung cancer stage 4. He is on weekly IV palliative chemotherapy. He visits his treating doctor saying that his chest pain has increased in severity even though he is having for last six months. When asked whether he has other part of body which he denies. He describes his pain as sharp as if like cutting by knife. He feels better when he takes paracetamol but does not get relief completely. Pain is worse during cough. O/E he is fully conscious and well oriented to time place and person. He takes his normal Nepali meal. When assessed by NRS, the score is 8. He thinks disease is the cause of pain.

Case Study 2
Sita Rai 50 years female diagnosed lady with breast cancer was on Herceptin injection. She has been teaching in school normally as before. Her appetite is ok and taking normal meal. She came to hospital with complains of backache. Pain was radiating to lower leg with burning and tingling feeling. She feels better with rest and Ibuprofen tablet. Her pain was exaggerated when she walks. Pain score is 7 on NRS. She thinks progression of disease may be the cause of pain.

Case Study 3
Mrs Babita Bari 40 years female diagnosed as liposarcoma of rectal 8 years back. She has gone under exploratory laparatomy with resection of tumor in 2013 and again in 2019. Now she is presented with pain at back since 5 months, radiating to B/L thigh and at abdomen since 2 months. Pain has increased in severity for 10 days for which she has taken tab aceclofenac BD for 5 days and feels better but doesn't get relief completely. She describes her pain as sharp at back and numbness and burning type in B/L thigh, cramping and gnawing type of pain in abdomen. When assessed by NRS, the score is 5 for back pain and 8 for abdomen pain.

Case Study 4
Dil Maya Soni 27 years female case of dumbbell neurofibroma, D10/D11 laminectomy and tumor excision done on 2018/03/14 and D10.D11 facetectomy with total excision of extra- axial neurofibroma with patch repair and wound exploration with repair on 2020/02/01. Currently she is admitted in hospital due to pain at back radiating to umbilicus for 1 month. Pain has increased in intensity for 13 days for which she has taken ketorolac for 3 days but doesn't get relief from pain. She describes her pain as cramping, tingling, and sharp when assessed by NRS (numeric rating scale) the score is 8.

Supplementary Table 3. Demographic variables considered in univariate modeling of overall SUS scores.

**Significance of covariates in univariate models of SUS**	
**Parameter**	**P-value**
Age*	<0.0001
Clinical role	0.0967
Institution*	0.0068
Primary current practice area	0.2037
Total years as nurse or physician*	0.0017
Formal training completed in palliative care and/or cancer pain management*	0.0078
Pre-existing familiarity with the NAPCare PMG	0.5352
Frequency of use of mobile apps to provide clinical care to patients	0.0216
Frequency of use of mobile apps for personal reasons*	0.0008

**significant at p<.10*

10.3389/fpain.2022.910995

Supplementary Table 4. Variables of significance in final multivariate SUS model.

Parameter	Estimate	Standard Error	P-value
**Intercept**	68.24	1.76	
**Age**			0.0124*
18 - 30 years old	4.19	3.34	
31 - 40 years old	7.46	2.61	
41 and older (ref)	0.00		
**Total years of experience**			0.0371*
5 years or less	0.85	3.03	
6 - 10 years	5.98	2.76	
More than 10 years (ref)	0.00		
**Formal training in palliative care**			0.0189*
No	3.96	1.66	
Yes (ref)	0.00		

**significant at p<.05*

Supplementary Table 5. Demographic variables considered in univariate modeling of overall MARS scores.

**Significance of covariates in univariate models of MARS**	
**Parameter**	**P-value**
Age	0.1274
Clinical role	0.0834*
Institution	0.0030*
Primary current practice area	0.4310
Total years as nurse or physician	0.1960
Formal training completed in palliative care and/or cancer pain management	0.7907
Pre-existing familiarity with NAPCare PMG	0.3490
Frequency of use of mobile apps to provide clinical care to patients	0.5086
Frequency of use of mobile apps for personal reasons	0.6285

**significant at p<.10*

Supplementary Table 6. Variable of significance in final multivariate MARS model.

**Final model of MARS: parameter estimates and significance**			
**Parameter**	**Estimate**	**Standard Error**	**P-value**
**Intercept**	3.91	0.08	
**Institution**			0.0030*
Hospice	0.39	0.17	
Private hospital, cancer	0.39	0.11	
Public hospital, cancer	0.11	0.11	
Public hospital, general	0.00		

**significant at p<.05*
